# *Trichoderma harzianum* enhances lettuce biomass and modulates plant-soil emerging contaminant dynamics under reclaimed wastewater irrigation

**DOI:** 10.1007/s10532-026-10291-0

**Published:** 2026-04-15

**Authors:** Monica Brienza, Juan Manuel Peña-Herrera, Vincenzo Trotta, Serge Chiron, Andrés Sauvêtre

**Affiliations:** 1 Dipartimento di Scienze di Base e Applicate, Università degli Studi della Basilicata, Via dell’ateno 10, 85100 Potenza, Italy; 2 HSM, Univ Montpellier, CNRS, IRD, Montpellier, France; 3 Laboratorio Bioanalitics, Vicerrectoría de Investigaciones, Universidad del Valle, Cali, Colombia; 4 Dipartimento di Scienze Agrarie, Forestali, Alimentari ed Ambientali, Università degli Studi della Basilicata, Via dell’ateno 10, 85100 Potenza, Italy; 5 HSM, Univ Montpellier, IMT Mines Alès, CNRS, IRD, Alès, France

**Keywords:** Reuse, Carbamazepine, Climbazole, Plant uptake, Transformation products, Phytohormones, Phytoremediation

## Abstract

The use of wastewater for irrigation in agricultural soils offers a sustainable means to reduce freshwater consumption and recycle nutrients, but also poses contamination risks associated with emerging pollutants. Furthermore, potentially toxic transformation products may form in soil and rhizosphere, and could subsequently be taken up by plants. Several products containing spores of symbiotic fungi are available and could serve as effective solutions to enhance the biodegradation of micropollutants in agricultural soils irrigated with treated wastewater. In this study, the effect of the fungus *Trichoderma harzianum* on the distribution of two emerging contaminants, carbamazepine and climbazole, and their main transformation products among soil and lettuce tissues was investigated under controlled conditions. Physiological effects were also investigated by quantifying phytohormones in roots and leaves. Inoculation with *Trichoderma harzianum* did not significantly affect the uptake of the parent compounds but increased the concentration of their transformation products in soil and reduced their levels in plant leaves after three weeks. The fungus enhanced plant biomass and altered certain phytohormones involved in defense mechanisms such as salicylic acid and microbiome recruitment in roots and soils. Findings of this study provide valuable insights that can be effectively applied to crop cultivation using reclaimed wastewater, leveraging readily available biological products for improved sustainable practices.

**Supplementary Information:**

The online version contains supplementary material available at 10.1007/s10532-026-10291-0.

## Introduction

The use of treated wastewater for irrigation in crop production is a promising solution against water scarcity that has been promoted in countries with increasing water supply problems to deal simultaneously with population growth, rapid urbanization, and climate change (Mishra et al. 2023). In May 2020, the EU adopted Regulation (EU) 2020/741 of 25 May 2020 establishing minimum requirements to promote the reuse of reclaimed water. Halleux (2019) projected that wastewater reuse would. Halleux, (2019) projected that wastewater reuse would increase increase from 1.1 billion cubic meters to 6.6 billion cubic meters by 2025. However, challenges arise regarding wastewater quality since treated wastewater may contain residues of contaminants such as pharmaceutical compounds (PhACs), since wastewater treatment plants (WWTPs) are not designed for the removal of these kinds of contaminants (Al-Hazmi et al. 2023). In addition, in remote or low-populated regions, wastewater only receives primary treatment processes consisting of sedimentation and natural aeration, which makes the occurrence of PhACs even higher (Montemurro et al. 2018). From this point of view, the consumption of contaminants, including PhACs, through food irrigated with reclaimed water has likely increased (Picó et al. 2018).

Studies about PhACs uptake by crops irrigated with wastewater or amended with sludge from WWTPs have shown an uptake and translocation of such compounds to the edible parts of the plant (Keerthanan et al. 2021; Manasfi et al. 2021; Christou et al. 2019). Following uptake, these molecules can disturb the basal plant metabolism, triggering stress responses and detoxification mechanisms in the plant (D’Alessandro et al. 2020; Kosakivska et al. 2021). Stress responses can involve variations in the phytohormone levels present in the plant, which are associated with the regulation and development of physiological processes within the individual as plant growth, germination, reproduction, development, and protection (Anjali et al. 2023). In this context, soil microorganisms like *Trichoderma* spp. can alleviate plant stress by contributing to the bioremediation of PhACs in agricultural soils (Yaashikaa et al. 2022)

The general principle of xenobiotic detoxification and the specific enzymatic reactions involved in xenobiotic metabolism have been extensively described using traditional pollutants such as pesticides and lately also with organic micropollutants (Moncrieffe et al. 2022; Pérez Solsona et al. 2021). However, there is limited understanding regarding the physiological response of plants following the uptake and metabolism of PhACs, as well as the characteristics and impacts of the resulting transformation products (TPs) (Wang et al. 2023a, b). Plant metabolism aids in detoxifying the entire plant, akin to the processes observed in mammalian systems (Malchi et al. 2022; Sauvêtre et al. 2021).

*Trichoderma* is a genus of fungi found ubiquitously in soils. Many *Trichoderma* species can colonize root surfaces and establish mutualistic relationships with plants (Poveda et al. 2020). *Trichoderma harzianum* has antifungal properties in crops and is used as a biocontrol agent (Mukhopadhyay and Kumar 2020). Some species act as endophytes, providing benefits such as enhanced growth, stress tolerance, and disease resistance (Chaudhary et al. 2022). *Trichoderma spp*. are among the first organisms to come in contact with micropollutants in wastewater, contributing to their transformation before plant uptake (Shahid et al. 2020). They can also affect plant membrane permeability, affecting plant uptake (Elkelish et al. 2020). *Trichoderma* has been shown to alleviate phytotoxicity related to arsenic uptake in lettuce plants, but no studies have addressed organic micropollutants (Khan et al. 2020). To characterize this tripartite *plant-Trichoderma-micropollutants* interaction, two PhACs were selected with different properties and behavior in soils and plants: carbamazepine (CBZ) and climbazole (CLB).

CBZ and CLB are compounds usually detected in wastewater effluent. CBZ with a logKow of 2.45 and found in neutral form in irrigation water (pH 7) is a reference compound in plant uptake and metabolism studies (Manasfi et al 2021; Sauvêtre et al 2021). CBZ phase I TPs generated in soils or in plant tissues, involve generally CYP450 enzymes (Sauvêtre et al 2021). Therefore, high concentrations in plant tissues can inhibit CYP450 regular functions such as biosynthesis of secondary metabolites and phytohormones (PHs) involved in plant defense and stress response. In contrast, CLB is more hydrophobic (logKow 3.46) and is partially protonated at pH 7 (pKa 7.5), therefore accumulating more in soil and plant roots (Manasfi et al 2021). In addition, as with all azoles, CLB inhibits CYP450 enzymes and potentially the synthesis of PHs (Dutta et al. 2023) and has been indicated to be of environmental concern based on phytotoxicity tests (Sochacki et al. 2021).

In this context, the present study evaluated (i) the *T. harzianum* effect on CBZ and CLB uptake levels in *Lactuca sativa* plants irrigated with reclaimed wastewater, (ii) the changes in the distribution of TPs in soils and plant tissues after inoculation with the fungus, and (iii) the physiological responses of lettuce plants to PhACs and *T. harzianum* through biomass and PHs analysis. To our knowledge, the combined effects of *T. harzianum* inoculation and irrigation with treated wastewater on the fate of CBZ, CLB, and their TPs in soil and plant systems with the associated plant physiological responses have not been investigated yet. This is the major contribution and originality of this work.

## Materials and methods

### Chemicals

PhACs and PHs analytical standards including deuterated analytical standards used as internal labeled standards (ILS) were > 98% purity and purchased from different commercial suppliers such as Sigma–Aldrich (St Quentin-Fallavier, France), Toronto Research Chemicals (Toronto, Canada), Santa Cruz Biotechnologies (TX, USA), LGC standards, (Middlesex, UK). Table 1 presents a complete list of chemical analytes used during experiments and analysis with molecular formula, molecular weight, monoisotopic mass, and structure. Each target analyte and ILS solution were produced in methanol (MeOH) at a concentration of 1 mg/mL, from which the necessary dilutions were prepared for enrichment and the preparation of the calibration curves. The ILS was added to the samples used as surrogate standards at 1 ng/µL. All solutions were stored at − 25 °C.

**Table 1:** Structures and analytical parameters for the quantification of PhACs and PHs in soils and lettuce plant tissues

ID	Analyte	Ionization mode	Molecular formula (M)	Rt (min)	Monoisotopic mass (g/mol)	m/z (M + H)^+^/(M-H)^−^	Structure
CLB	CLB	+	C_15_H_17_ClN_2_O_2_	6.7	292.0978	293.1051	
CBZ	CBZ	+	C_15_H_12_N_2_O	7.1	236.0950	237.1022	
JA	Jasmonic acid	+	C_12_H_18_O_3_	7.1	210.1256	211.1329	
IAA	Indole-3-acetic acid	+	C_10_H_9_NO_2_	7.1	175.0633	176.0706	
GA1	Gibberellin A1	−	C_19_H_24_O_6_	5.8	348.1573	347.1500	
GA3	Gibberellin A3	−	C_19_H_22_O_6_	5.7	346.1416	345.1343	
GA4	Gibberellin A4	−	C_19_H_24_O_5_	7.7	332.1624	331.1551	
GA7	Gibberellin A7	−	C_19_H_22_O_5_	7.6	330.1467	329.1394	
SA	Salicylic acid	−	C_7_H_6_O_3_	6.0	138.0317	137.0244	
ABA	Abscisic acid	−	C_15_H_20_O_4_	6.95	264.1361	263.1289	

For the extraction process and LC–MS/MS analysis, the following solvents were used: Ultra-pure water (UPW) obtained using a Millipore system; acetonitrile (ACN) HPLC-grade, and MeOH HPLC-grade were purchased from Carlo Erba Reagents S.A.S. (Val de Reuil, France). Formic acid (FA) puriss p.a. ACS reagent analytical grade was purchased from Sigma–Aldrich (Steinheim, Germany), acetone was purchased from Honeywell Riedel-de-Haën (Seelze, Germany).

For the EDTA-McIIvaine buffer, di-sodium hydrogen phosphate dihydrate (Na_2_HPO_4_·2H_2_O) and citric acid monohydrate (C_6_H_8_O_7_·H_2_O) were obtained from Merck (Darmstadt, Germany) and anhydrous ethylenediamine tetraacetic acid (EDTA) (≥ 99%) from Sigma-Aldrich.

### Growth chamber experiments

Lettuce plantlets (*Lactuca sativa* var. *Batavia*) were grown in pots containing a synthetic soil mix (35% peat, 10% clay soil, 25% sand, and 30% perlite). Plants were irrigated with tap water under controlled conditions in a growth chamber (Binder KBWF 720) for two weeks to adapt to the growing conditions. These were set to 16 h of light, 16–25 °C and a relative air humidity of 60%. Each plant was grown in a 9 × 7x7 cm pots and pots were divided into three trays containing each a set of 9 plants. The 1st tray (control) contained pots irrigated with wastewater. The 2nd tray contained pots irrigated with spiked wastewater. The 3rd tray contained inoculated pots with *T. harzianum* and irrigated with spiked wastewater. In all cases, wastewater was autoclaved at 121 °C for 20 min to reduce the impact of wastewater-borne microbes on pharmaceutical metabolism. Wastewater was collected after secondary treatment based on three successive lagoons from Murviel-lès-Montpellier, France.

Inoculation with *T. harzanium* was performed using the commercial product Canna AkTRIvator®, purchased from Canna International BV (Breda, the Netherlands). Following the recommendations of the manufacturer, inoculation was done by mixing 1 g of powder (dried spores, 1.15% w/w) per kg of soil and diluting 1 g of powder per liter of irrigation water during the potting, adding 100 mL to each pot. Wastewater was spiked with CBZ and CLB at a final concentration of 200 µg/L, a concentration representative of real (worst) cases in WWTPs and allowing the identification of TPs in the experimental conditions. Plants were irrigated to reach the soil water holding capacity (WHC). Afterward, plants were irrigated again when the soil reached approximately 60% of the measured WHC. Daily irrigation and cumulative volume and amount of PhACs are indicated in Table S1.

### Sample treatment

Three plants were harvested after 1, 2 and 3 weeks of treatment from each tray. Plant tissues were separated into roots and aerial edible tissues. Stems represented a very limited fraction of aboveground biomass and were therefore not separated from leaves. Plant root tissues were then cleaned with tap water to remove excess attached soil from roots, weighted, immediately frozen in liquid nitrogen and stored at – 80 °C. Samples were then freeze-dried, grounded using a ball mill and kept in the freezer at – 20 °C until the extraction. Soil samples were stored in alumina boxes and kept in the freezer at − 80 °C until extraction.

### Analytes extraction

Analytes extraction from lettuce leaf samples was performed using a QuEChERS method as previously reported (Montemurro et al. 2020). In summary, 1 g of freeze-dried and milled lettuce was rehydrated with 9 mL of UPW, vortexed for 1 min and left to settle for 1 h. The ILS mixture was then spiked with 50 µL of 1 ng/µL, vortexed again and left to settle for 1 h. Subsequently, analytes were extracted with a mixture of 10 mL of ACN and 50 µL of FA. After vortexing for 1 min, the extracting salts from the QuEChERS kit were added (4 g MgSO_4_ + 1 g NaCl, Agilent Technologies, CA, USA). Vigorous manual shaking was performed for 30 s and the sample was then vortexed for 1 min. The samples were centrifuged for 10 min at 4 °C and 4000 rpm. After being collected into test tubes, the supernatant was refrigerated for the whole night. The next day, a dispersive type purification (d-SPE) was performed with cleaning salts Supel QuE kit (Sigma-Aldrich) consisting of 150 mg PSA, 150 mg C_18_, and 900 mg MgSO_4_. To do this, the salts were added to 6 mL of the supernatant, manually shaken for 30 s and then vortexed for 1 min. The samples were centrifuged at 4000 rpm at 4 °C for 10 min. Finally, 1 mL of the extract was evaporated with a stream of nitrogen and reconstituted in 1 mL with H_2_O:MeOH 98:2. The reconstituted mixtures were filtered through 0.2 µm PTFE filters (Macherey–Nagel GmbH & Co. KG, Düren, Germany). The extraction was used to follow the target PhACs and simultaneously, the PHs present in the lettuce plants.

Analytes extraction from root samples was carried out using a method from (Sunyer-Caldú and Diaz-Cruz 2021). In summary, for the analysis, 0.2 g of freeze-dried roots of lettuce was rehydrated with 1.8 mL of EDTA McIlvaine buffer solution, vortexed for 1 min and left to settle for 1 h. The mixture was then spiked with 10 µL of 1 ng/µL of ILS solution, vortexed again and left to settle for 1 h. Subsequently, analytes were extracted with 2 mL of ACN. After 1 min of the vortex, 1.0 g of the extracting mixture salts from the QuEChERS kit were added. Manual shaking was performed for 30 s and then vortexed for another 1 min. The samples were centrifuged at 4000 rpm, at 4 °C for 10 min, then 0.2 mL of the supernatant was evaporated with a stream of nitrogen and reconstituted in 0.2 mL of H_2_O:MeOH 98:2.

Analytes were extracted from soil samples using an method described by Manasfi et al. (2021). Soil samples were homogenized before extraction, and subsamples were produced by coning and quartering. Afterwards, 10 g of soil was placed in a 50 mL falcon and 3 mL of acetone was added. The sample was spiked with 50 µL of 1 ng/µL ILS solution. The samples were vortexed and left to settle at room temperature for one night, to allow the evaporation of the solvent. The next day, samples were hydrated using 8 mL of EDTA-Mcllvaine buffer, vortexed, and settled for 1 h. Thereafter, 10 mL of ACN were added to the sample and vortexed. The extracting salts (QuEChERS Orig Meth kit) were added, immediately hand shaken and then vortexed for 1 min. Finally, the samples were centrifuged at 4000 rpm, at 4 °C for 10 min, then 1 mL of the supernatant was evaporated with a stream of nitrogen and reconstituted in 1 mL of H_2_O:MeOH 98:2.

### Chromatographic separation and LC–MS/MS analysis

For analysis of target analytes, TPs and PHs, an LC–MS/MS system was used. Chromatographic separation was achieved using an HPLC Vanquish Autosampler system split sampler with a quaternary pump (Thermo Fisher Scientific, Les Ulis, France). A packed column EVO C18 KINETEX reversed-phase (50 × 2.1 mm, 2.6 µm particle size, Phenomenex, Torrance, CA) thermostated at 30 °C was used. The mobile phases were (A) H_2_O + 0.1% FA and (B) MeOH at a flow rate of 0.3 mL/min. The injection volume was 10 µL with the autosampler temperature set to 5ºC. The chromatographic separation was conducted using a gradient elution protocol, which involved the following solvent compositions and corresponding run times: 40% for 2.5 min, 50% for 4.0 min, 80% for 5.0 min, 80% for 7.0 min, 98% for 7.1 min, 98% for 8.0 min, 2% for 8.5 min, and 2% for 11 min.

The divert valve was activated to flow the solution to the waste between 0–1 min and 10–11 min. The mass spectrometric analysis was performed in a Q-Exactive™ Focus Orbitrap (Thermo Fisher Scientific) equipped with an electrospray source and operated in positive/negative ionization mode in full scan acquisition (70,000 FWHM resolution at m/z 200) and PRM acquisition (35,000 FWHM resolution at m/z 200) mode from 100 to 600 m/z scan range setting automatic gain control target at high dynamic range (1e6) and the maximum injection time of 50 ms. Data processing was carried out using instrumental software Xcalibur 4.2.47 (Thermo Fisher, Scientific, San Jose, USA). The Heated electrospray (HESI) conditions for positive mode analysis of CBZ, CLB, jasmonic acid (JA), indole-3-acetic acid (IAA) and CBZ and CLB metabolites, were: sheath gas flow rate of 35 (arbitrary units, AU), spray voltage of 3.5 kV, auxiliary gas flow rate of 10 (AU), sweep gas flow rate of 2 (AU), capillary temperature 320 °C and auxiliary gas heater temperature 300ºC. In the case of negative mode analysis of giberellins GA1, GA3, GA4, GA7 (GA1, GA3, GA4, GA7), abscisic acid (ABA) and salicylic acid (SA) the conditions were sheath gas flow rate 50 (AU); auxiliary gas flow rate 5 (AU); sweep gas flow rate 2 (AU); spray voltage 3.0 kV, capillary temperature 320 °C and auxiliary gas heater temperature 330ºC. The previous conditions were adapted from previous publications of Chen et al. 2016 and Manzi et al. 2015.

### Quantitative analysis

For the quantification of PhACs and PHs, matrix calibration curves were made according to the matrix match standard procedure using the internal standard method (Commission Regulation EU 2020/741), in ranges presented in Table 1. Indole Acetic acid d_2_ (IAA-d_2_) and abscisic acid d_6_ (ABA-d_6_) were used as labeled internal standards for quantification in positive and negative modes respectively. Table 1 presents some analytical parameters for the quantitative analysis of the compounds and their TPs.

### Qualitative analysis.

A tentative list of TPs for CBZ and CLBwas prepared and then all samples of leaves, roots and soil were analyzed by LC–MS/MS. The suspect candidates were determined according to the exact mass m/z, the error of mass accuracy lower than 5 ppm, retention time, peak areas and fragmentation pattern. To confirm the presence of TPs, MS^2^ analysis was conducted, comparing the retention time and fragmentation profiles with those obtained from analytical standards under identical conditions (Table 2).

**Table 2:** Structures and analytical parameters of CBZ and CLB transformation products (TPs) identified in soils and lettuce plant tissues exposed to CBZ and CLB

ID	Analyte	Molecular formula (M)	Rt (min)	Monoisotopic mass (g/mol)	m/z (M + H)^+^	Structure	Fragments and relative abundance
CBZ-epox	CBZ-10,11-epoxide	C_15_H_12_N_2_O_2_	6.3	252.0899	253.0982		180.0814 (100%) 167.0736 (18.30%) 181.0850 (15.5%) 152.0626 (9.85%)
CBZ-diOH (cis)	CBZ-cis-10,11-dihydroxy	C_15_H_14_N_2_O_3_	6.0	270.1004	271.1089		180.0815 (100%) 167.0737 (16.38%) 181.0849 (16.34%) 179.0738 (9.65%)
CBZ-diOH (trans)	CBZ-trans-10,11-dihydroxy	C_15_H_14_N_2_O_3_	6.0	270.1004	271.1089		180.0815 (100%) 167.0737 (16.38%) 181.0849 (16.34%) 179.0738 (9.65%)
CBZ-10OH	CBZ-10-OH	C_15_H_14_N_2_O_2_	6.2	254.1055	255.1141		193.0894 (100%) 192.0815 (56.52%) 179.0937 (53.53%) 165.0765 (30.07%)
CBZ-3OH	CBZ-3-OH	C_15_H_12_N_2_O_2_	6.6	252.0899	253.0982		167.0737 (100%) 180.0816 (57.32%) 209.0844 (19.97%)
ACRI	Acridine	C_13_H_9_N	5.1	179.0735	180.0813		152.0625 (100%) 178.0658 (80.93%) 169.0654 (45.11%)
ACRO	Acridone	C_13_H_9_NO	6.8	195.0684	196.0764		196.0765 (100%) 197.0799 ( 16.07%)
IMI	Iminostilbene	C_14_H_11_N	8.0	193.0891	194.0974		191.0737 (100%) 192.0813 (91.43%) 165.0705 (71.05%) 134.0606 (31.93%)
CLB-OH1	CLB-OH1	C_15_H_19_ClN_2_O_2_	6.00	294.1135	295.1221		69.0450 (100%) 139.0057 (19.25%)
CLB-OH2	CLB-OH2	C_15_H_19_ClN_2_O_2_	6.36	294.1135	295.1221		69.0451 (100%) 285.3877 (9.45%)

### Calculation of bioconcentration, root concentration and translocation factors

To investigate the uptake and translocation of target compounds within the lettuce plants, the Bioconcentration Factor (BCF), Root Concentration Factor (RCF), and Translocation Factor (TF) were determined. These parameters were computed using the subsequent formulas:

1 $$ Bioconcentration Factor \left( {BCF} \right) = \frac{{\left[ {Concentration in Leaves} \right] }}{{\left[ {Concentration in Soil} \right]}} $$

2 $$ Root Concentration Factor \left( {RCF} \right) = \frac{{\left[ {Concentration in Roots} \right] }}{{ \left[ {Concentration in Soil} \right]}} $$

3 $$ Translocation Factor \left( {TF} \right) = \frac{{\left[ {Concentration in Leaves} \right] }}{{/ \left[ {Concentration in Roots} \right]}} $$

### Statistical analyses

Leaf biomass and concentrations of carbamazepine (CBZ) and climbazole (CLB) in leaves, roots, and soils were analyzed separately using two-way analysis of variance (ANOVA). The factors included Treatment (Ls, Ls + PhACs, and Ls + PhACs + Th) and Week (week 1, week 2, week 3). For each variable, the effects of treatment, week, and their interaction were tested. When ANOVA indicated significant effects (p < 0.05), Tukey’s honestly significant difference (HSD) post-hoc tests were applied to identify pairwise differences between treatments. In cases where the Treatment × Week interaction was significant, post-hoc comparisons were conducted within each week. A significance level of p < 0.05 was used for all tests.

To fully exploit the multivariate nature of the dataset, multivariate analyses, including principal component analysis (PCA) and a two-way multivariate analysis of variance (MANOVA) based on permutations, were conducted in addition to ANOVA, to explore relationships among biomass, parent compounds, transformation products, and phytohormones, and to evaluate whether CBZ, CLB, and *Trichoderma* treatments influenced the observed patterns. Analyses were performed using log-transformed values [log(x + 1)]. PCA and MANOVA were performed on centered and standardized variables (correlation matrix) and were conducted separately for leaf, root, and soil compartments. Biomass was excluded from the soil PCA, as it was not measured for this compartment.

## Results and discussion

### Biomass of exposed lettuce plants

**Fig. 1:**
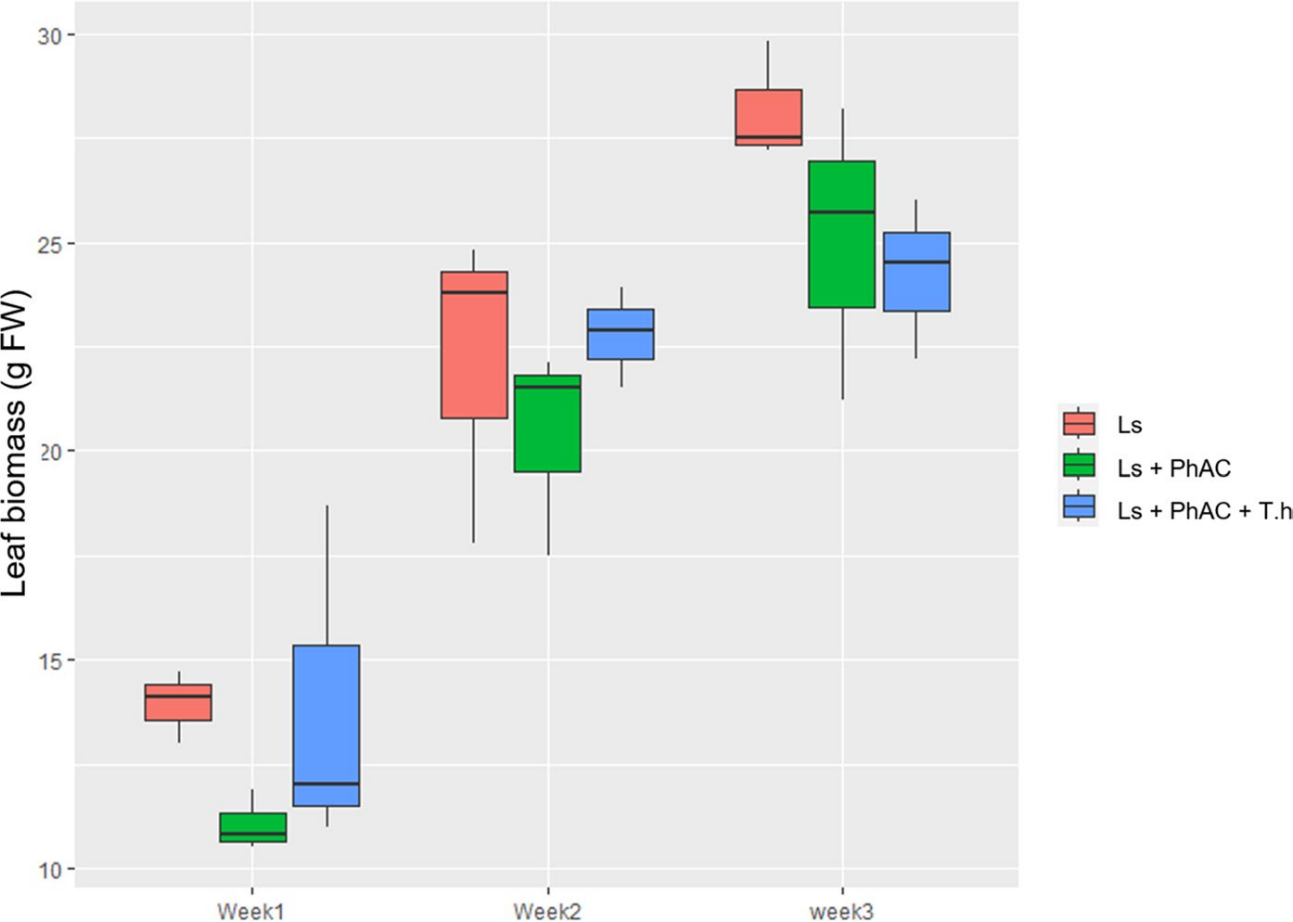
Leaf biomass in g (FW) measured in control plants irrigated with wastewater (Ls), plants irrigated with wastewater spiked with 200 µg/L CBZ and CLB (Ls + PhAC) and plants inoculated with T. harzianum and irrigated with wastewater spiked with 200 µg/L CBZ and CLB (Ls + PhAC + Th), n = 3

Root fresh biomass was quantified. No consistent treatment-dependent trend in root biomass was observed across time points, likely reflecting high variability associated with partial tissue loss during harvest (Fig. S1). Biomass responses were evaluated on a leaf fresh-weight basis, as leaves represent the harvested and commercial part of lettuce and are therefore the most relevant from an agronomic perspective. Leaf biomass was measured in lettuce plants exposed to PhACs for three weeks (Fig. 1). Overall, control plants exhibited a higher biomass than plants exposed to PhACs, with an average daily production of 1.64 g FW (Ls). When irrigated with wastewater spiked with CBZ and CLB, the production was reduced to 1.41 g FW day^−1^ (Ls + PhAC). In previous studies, CLB and other azoles exhibited growth-retarding symptoms presumably related to an interference with phytohormone synthesis as known for structurally similar fungicides used in agriculture (Richter et al. 2016). Inoculation with *T. harzianum* had a positive effect in treated plants resulting in an average biomass production of 1.59 g FW day^−1^ (Ls + PhAC + Th). The control group (Ls) showed the highest fresh weight throughout the three weeks with aerial biomass of 13.9, 22.1 and 28.2 g FW after 1, 2 and 3 weeks respectively (Fig. 1), whereas the plants irrigated with treated wastewater spiked with PhACs (LS + Ph) showed a lower growth rate (11.1, 20.4 and 25 g FW). Plants irrigated with treated wastewater spiked with PhACs and inoculated with *T. harzianum* (LS + PhAC + Th) showed a growth rate similar to that of the control group in the first and second weeks, but a slightly lower growth rate in the third week. Although plant tissues were lyophilised prior to chemical analyses, dry-weight biomass could not be reliably quantified due to material loss during freeze-drying. To minimize variability related to tissue water content, plants were irrigated when soil moisture reached 60% of water-holding capacity, thereby limiting differences in hydration status among treatments and supporting the use of fresh-weight–based comparisons. Overall, these results suggest that the presence of PhACs in treated wastewater could negatively impact the growth of lettuce plants, but the presence of *T. harzianum* might have a positive effect on plant growth during the first two weeks of exposition. However, only statistically significant differences were observed between control plants and exposed inoculated plants (LS + PhAC + Th) in the third week, due to a high variability between individuals in the same group. *Trichoderma* spp. are able to promote plant growth through the synthesis of PHs and phytoregulators or nutrient solubilization (Tyśkiewicz et al. 2022). The stagnation in biomass increase observed in the third week could be related to a higher metabolism of PhACs in the soil attributed to the presence of *T. harzianum* (fungus metabolism) contributing to a higher accumulation of potential toxic TPs in the plant tissues over time (Jha et al. 2023). The scientific literature on plant exposure to PhACs has mainly focused on plant uptake and to a lesser extent on phytotoxicity effects (Wei et al. 2023). While some studies have reported a reduction of plant biomass after exposure to PhACs (Alkimin et al. 2019) in other studies physiological changes such as photosynthesis reduction and metabolic changes are not accompanied by biomass reduction (Mascellani et al. 2023). The experiments showed, that apart from a reduction in biomass, no visible or apparent damage to the plants was observed following exposure to PhACs. Consequently, it was not possible to establish phytotoxic effects based on the observed parameters.

### Fate of pharmaceuticals in plants and soils

**Fig. 2:**
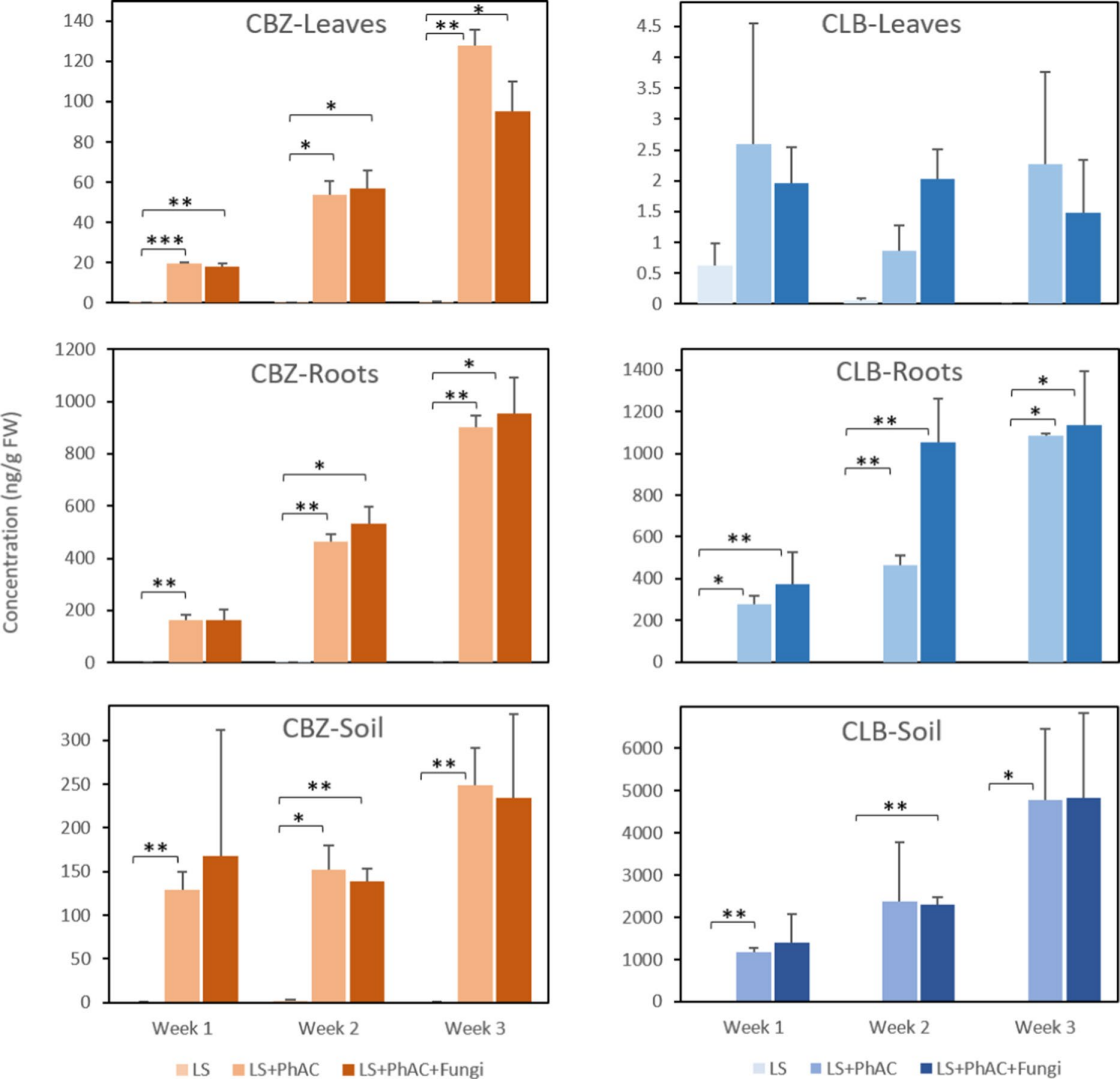
Concentrations of carbamazepine (CBZ) and climbazole (CLB) in control plants irrigated with wastewater (Ls), plants irrigated with wastewater spiked with 200 µg/L CBZ and CLB (Ls + PhACs) and plants inoculated with T. harzianum and irrigated with wastewater spiked with 200 µg/L CBZ and CLB (Ls + PhACs + Th), n = 3

In general, neutral compounds with a logK_ow_ between 1 and 3 are subjected to plant uptake and accumulation in aerial parts, while more hydrophobic or polar compounds as well as charged compounds accumulate in soils and roots, adsorbed onto soil particles or stored in interstitial and intercellular free water as a result of their charge and their interaction with soil particles and biological membranes (Bigott et al 2021; Collins et al 2011). Therefore, CBZ (logK_ow_ = 2.45 and neutral at pH 7) and CLB (logK_ow_ = 3.46 and partially protonated at pH 7) were selected for their different expected behavior in soil–plant systems. CBZ and CLB were detected and quantified in soils, roots and leaves of plants irrigated with wastewater, at concentrations ranging from less than one ng/g FW to 1.14 µg/g FW in plant tissues and between 1 ng/g FW to 4.83 µg/g FW in soils (Fig. 2). The presence of both compounds in control samples was observed at values lower than 1 ng/g FW, which indicates negligible values for the effect of the study.

CBZ was found in soils and plant tissues irrigated with spiked wastewater.

In plants, a linear CBZ accumulation over time was observed in roots and leaf tissues. Maximum values were observed after three weeks in the leaves of exposed plants (128 ng/g) and in the roots of exposed and inoculated plants (953 ng/g). A less pronounced trend was observed in soils. After three weeks, plants exposed to PhACs and inoculated with *T. harzianum* demonstrated a slightly higher CBZ concentration in roots but lower in leaves and soils compared to the Ls + PhACs group (953 against 902 ng/g and 95 against 128 ng/g, respectively).

CLB accumulated in soils and roots over time, reaching 4832 ng/g in soils and 1137 ng/g in roots while the concentrations in leaf tissues were almost negligible (Fig. 2). After three weeks, inoculated plants were able to accumulate a slightly higher concentration in roots with 1137 against 1083 ng/g. Retention of CLB was also slightly higher in soils inoculated with the fungus, with 4832 against 4772 ng/g. However, no significant differences could be established between these two groups.

BCF, RCF and TF are key parameters used to assess the uptake of PhACs in plants. BCF indicates the potential for PhACs accumulation in plant biomass. RCF provides insights into the initial uptake by roots. TF shows the potential for translocation from roots to aerial plant parts. Understanding these uptake mechanisms is crucial for evaluating the environmental fate and potential risks associated with PhACs contamination in agricultural systems. BCF, RCF and TF were determined after three weeks (Table 3). CBZ exhibited higher RCF, BCF and TF (3.70, 0.53 and 0.14 respectively) compared to CLB (0.26, 3.99E^−04^ and 1.95E^−03^ respectively). While inoculation of *T. harzianum* resulted in a decrease of CBZ BCF and TF (- 11% and—28% respectively) and an increase of CBZ RCF (+ 27%), it did not affect significantly CLB RCF (+ 4%), and especially BCF and TF, as the obtained values are almost negligible.

**Table 3:** Root concentration factor (RCF), bioconcentration factor (BCF) and translocation factor (TF) after three weeks in lettuce plants irrigated with wastewater spiked with carbamazepine (CBZ) and climbazole (CLB) at 200 µg/L (Ls + PhAC) and lettuce plants irrigated with wastewater spiked with CBZ and CLB at 200 µg/L and inoculated with *T. harzianum* (Ls + PhAC + Th). Values are presented as mean ± standard deviation (n = 3)

		Ls + PhAC	Ls + PhAC + Th	**Δ**
CBZ	RCF	3.70 (± 0.70)	4.70 (± 2.60)	** + 27%**
	BCF	0.53 (± 0.11)	0.47 (± 0.24)	− 11%
	TF	0.14 (± 0.003)	0.10 (± 0.03)	−** 28%**
CLB	RCF	0.25 (± 0.12)	0.26 (± 0.10)	** + 4%**
	BCF	3.99E-04 (± 4.5E-04)	4.44E-04 (± 5.9E-04)	** + 11%**
	TF	1.95E-03 (± 1.8E-04)	1.32E-03 (± 1.5E-03)	− 32%

Results show that neutral compounds such as CBZ tend to be more concentrated in leaves compared to cationic compounds, consistent with previous research. Specifically, the results align with earlier findings showing a higher concentration of CBZ in lettuce leaves compared to CLB, in an experiment with lettuce plants irrigated for six weeks with spiked wastewater at 10 µg/L (Manasfi et al. 2021). Research by (Sochacki et al. 2021) revealed that CLB accumulates in the roots of the macrophyte *Glyceria maxima*, but no translocation was observed. TF observed in these experiments was 0.1 but the removal was near 90% indicating soil retention and metabolism in the rhizosphere rending mainly reduced CLB-alcohol. The removal in unplanted columns was only 56%. (Manasfi et al. 2021) reported a CBZ concentration of 660 ng/g in lettuce leaves after six weeks of irrigation with spiked wastewater at 10 µg/L, representing the highest accumulation among the 14 compounds analyzed. Conversely, CLB was nearly undetected in plant leaves, indicating poor uptake, with a resulting BCF estimated to be negligible.

This study demonstrated an increase in CBZ concentration in soils, from 130 ng/g in the first week to 248 ng/g after three weeks in plants irrigated with spiked wastewater. Interestingly, similar trends were observed in inoculated plants, but CBZ concentration was slightly lower (235 ng/g after three weeks). In addition, after three weeks, plants exposed to PhACs and inoculated with *T. harzianum* demonstrated a slightly CBZ higher concentration in roots (953 vs 901 ng/g) but lower in leaves (95 vs 128 ng/g), indicating that *T. harzianum* may have some impact on modifying the uptake and distribution of PhACs in the soil–plant system at long term.

In roots, CBZ was found in increasing concentrations from the first to the third week. Concentrations were lightly superior in inoculated plants. Similar behavior was observed for CLB with 1137 ng/g in the roots of inoculated plants against 1083 ng/g in exposed roots. The hypothesis that *T. harzianum* enhances CBZ and CLB uptake in roots may be supported by several factors. One potential explanation could be the presence of a larger specific surface area resulting from the fungal mycelium, which may increase the contact area between the pharmaceutical and the plant roots, thereby facilitating uptake (Malik et al. 2020). Additionally, *T. harzianum* may induce minor wounds or changes in the root structure, which could enable the penetration of CBZ into the root tissues (Adeola et al. 2022). This hypothesis aligns with previous studies suggesting that *Trichoderma* can enhance nutrient uptake and modify root morphology (Stewart and Hill 2014). The results indicate that *T. harzianum* may have a mitigating effect on CBZ accumulation in the leaves, as well as its retention in the soil. These findings suggest the potential of *T. harzianum* in modifying the fate of PhACs in wastewater-irrigated systems, highlighting its role in environmental remediation (Al-Farsi et al. 2018).

### Distribution of transformation products in soil and plant tissues

**Fig. 3:**
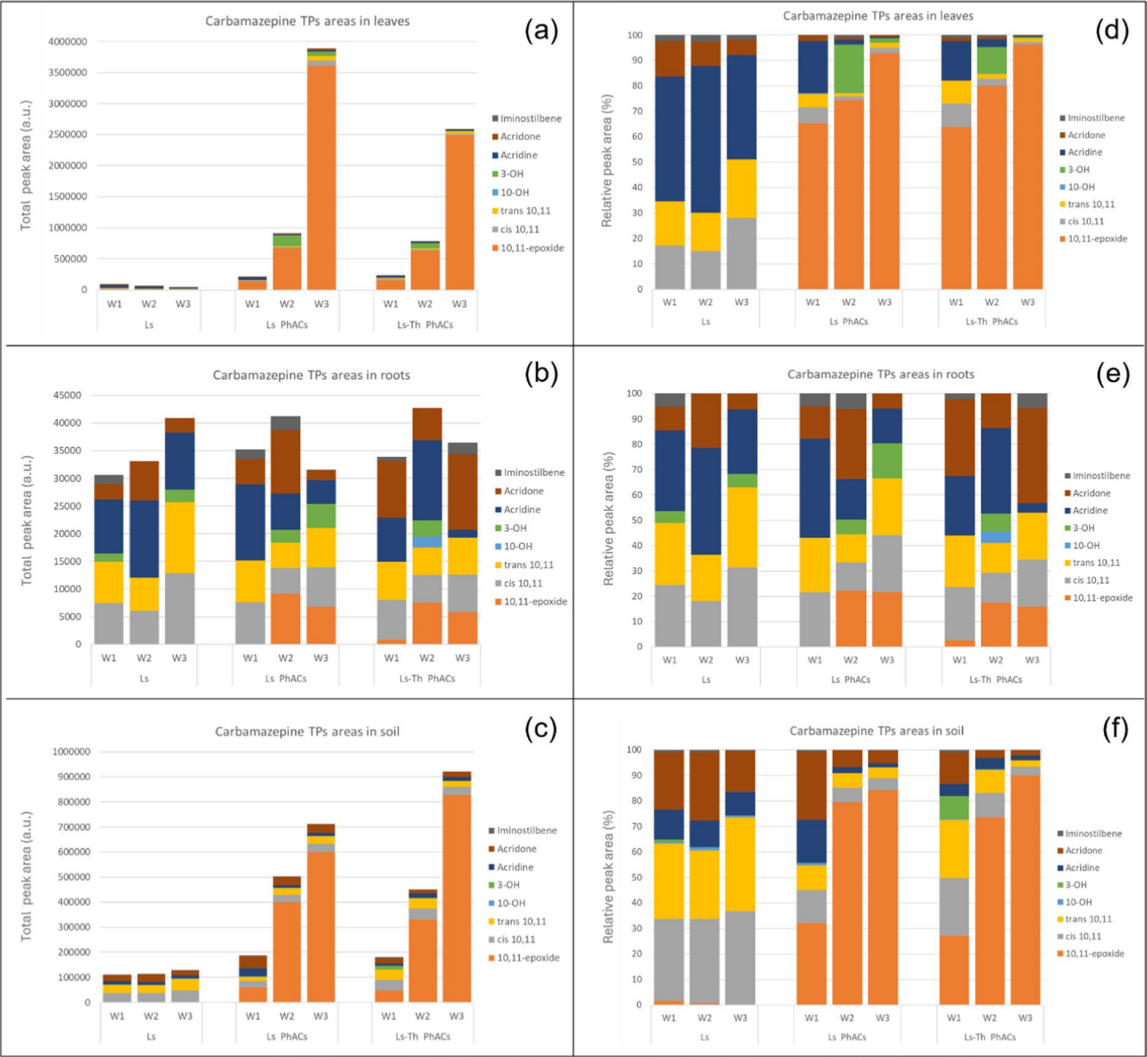
Distribution of carbamazepine (CBZ) transformation products (TPs) after three weeks of irrigation in plant tissues and soils of control plants irrigated with wastewater (Ls), plants irrigated with wastewater spiked with 200 µg/L CBZ and climbazole (CLB) (Ls + PhAC) and plants inoculated with T. harzianum and irrigated with wastewater spiked with 200 µg/L CBZ and CLB (Ls + PhAC + Th). Results are the average of three biological replicates and are expressed as LC–MS/MS peak areas (a, b, c) and as relative fractions based on LC–MS/MS peak areas (d, e, f)

After uptake, parent compounds can be metabolized in plant tissues, in addition to TPs formation in wastewater and soil. Due to TPs potential toxicity, their analysis is significant for a better risk assessment. To study the impact of *T. harzianum* on CBZ and CLB metabolism and accumulation of TPs, suspect screening was performed using a tentative list of hypothetical TPs. Eight TPs were identified for CBZ and two for CLB (all phase I metabolites), by comparison of MS^2^ spectra and retention times with those of their respective analytical standards (Table 2). Kinetics of identified TPs were determined in soils, roots and leaves by semi-quantification using peak areas (Fig. 3 and Fig. S1). Identified CBZ TPs included acridine, CBZ-10,11-epoxide, cis-10,11-dihydroxyCBZ, trans-10,11-dihydroxyCBZ, CBZ-10-OH, CBZ-3-OH, acridone, and iminostilbene (Table 2).

Across all experimental groups (Ls, Ls + PhACs, and Ls + PhACs + Th) and sampling weeks, TPs of CBZ were consistently detected in leaves, indicating their uptake and/or metabolism in plant tissues. In general, the proportion of TPs was much lower than CBZ in exposed plants, accounting for 2% in leaves and 0.5% in roots and soils. In control plants, this ratio was higher, reaching 9% in leaves, 64% in roots and 17% in soil after three weeks. CBZ TPs concentrations varied among experimental groups and sampling weeks, but increasing over time, as a whole. Figure 3.d presents the percentage distribution of TPs in leaves. Across all weeks, the predominant TP observed was CBZ-10,11-epoxide for Ls + PhACs, and Ls + PhACs + Th, resulting from CYP450 and peroxidase-mediated oxidation enzymes activity (Sauvêtre et al. 2018). Consequently, *T. harzianum* influenced the concentration of CBZ TPs in leaves without altering their chemical identity, confirming the observation of Mordechay et al. 2018. CBZ-TPs were also consistently detected in roots, indicating their uptake and accumulation within the plant root tissues. Figure 3.e illustrates the percentage distribution of TPs in roots. Throughout the three weeks, TPs were almost regularly distributed albeit varying in their distribution whereas there is no predominant one. The results about the distribution of CBZ-TPs in soil samples revealed CBZ-TPs persistence and accumulation in the soil matrix (Fig. 3.c). Same as leaves and roots, the presence of *T. harzianum* in the Ls + PhACs + Th group influenced the distribution of CBZ-TPs. The predominant TP observed in soil samples (Fig. 2.f) was again CBZ-10,11-epoxide for Ls + PhACs, and Ls + PhACs + Th, whereas it was absent for Ls, suggesting CBZ degradation as the main source. Acridone and Acridine, which are TPs resulting from subsequent oxidation or hydrolysis reactions showed significant percentages, while CBZ-10-OH, CBZ-3-OH, and Iminostilbene were detected in relatively lower percentages. The highest TPs concentrations were found in leaves while root tissues had the lowest concentrations, remaining almost stable, which could be explained by restricted enzymatic activity or effective translocation mechanisms (Brunetti et al. 2021). *T. harzianum* affected CBZ transformation pathways in both soil and plant tissues, most likely because of changing the microbial community structure or adding extra enzymes such as strain MHT1134 (Mao and Jiang 2021; Wang et al. 2024). *Trichoderma* spp. have been traditionally considered as soil fungi with saprophytic and epiphytic activities, thus colonizing mainly superficial layers of root surface and in some cases, gaining entrance into the plant (Harman et al. 2004). Some strains including *T. harzianum* have been reported to be endophytic (Chacon et al. 2007). This lifestyle would explain by one hand the increase of CBZ TPs in soils of inoculated plants, as CBZ is taken up by the mycelia and metabolized by fungal enzymes (some of the them such as laccases might be secreted to the extracellular surrounding soil) and by the other hand, the promotion of plant growth and defense mechanisms through a close relationship with its plant host.

The distribution of CLB-TPs was also investigated in leave, root, and soil. Only one TP has been detected, CLB-alcohol (CLB-OH) due to the reduction of the ketone under microbial degradation leading to the formation of two diastereoisomers, easily separated on a C-18 analytical column: CLB-OH1 (first eluting compound) and CLB-OH2 (second eluting compound) but both with the same m/z = 295 (Brienza and Chiron 2017). CLB-OH1 and CLB-OH2 were both generated in soil (see Fig. S1) even though CLB-OH2 predominated over CLB-OH1, most likely due a known stereoselective microbial reduction process and/or a faster degradation of CLB-OH1 over CLB-OH2. There was evidence of stereoselective uptake of CLB-OH in lettuce accounting for the only detection of CLB-OH2 in root. This result is consistent with the behavior of CLB-OH in constructed wetland using *Phragmites australis* (Sochacki et al. 2021). Stereoselective uptake by plant is a common process which has been documented for pesticides and pharmaceuticals (Wang et al. 2023a, b). However, inoculation with *T. harzianum* reduced RCF and TF values of CLB-OH2, limiting its bioaccumulation. Higher TF value of CLB-OH over CLB could be explained by its higher hydrophilicity while new trace CLB-OH1 detection in leaves might only reflect slightly higher concentrations than in roots that could be detected.

### Phytohormonal responses to pharmaceuticals

One of the PGPR traits most studied in *Trichoderma* spp. used in biocontrol is the synthesis of PHs involved in growth and developmental processes (Abdenaceur et al. 2022). Based on their primary functions, PHs can be categorized as growth-promoting hormones (such as IAA and gibberellins) and defense-associated hormones (like SA and ABA). *T. harzianum* has been demonstrated to stimulate the production of IAA, gibberellins, and several other PHs in a wide range of plant species (Martínez-Medina et al. 2011). These plant growth-promoting hormones play crucial roles in various physiological processes, including seed germination, root development, and stress responses, thereby enhancing plant growth and resilience (Sabagh et al. 2021). The fungus enhances plant stress tolerance through multiple mechanisms, including the PHs activation, the production of osmolytes, and the improvement of the redox-enzymatic machinery (Contreras-Cornejo et al. 2024). Given these observations, this study aimed to explore the impact of PhACs and *T. harzianum* on phytohormonal processes determining growth and development of lettuce plants.

PHs were analyzed in plant tissues. IAA, ABA, SA and gibberellins GA1, GA3, GA4 and GA7 were found at concentrations ranging from 0.02 to 97 ng/g (Fig. S2). JA was not detected. In leaves, only gibberellins were found, at concentrations below 1 ng/g for GA1, GA3 and GA4, and from 1 to 3 ng/g for GA7.

**Fig. 4:**
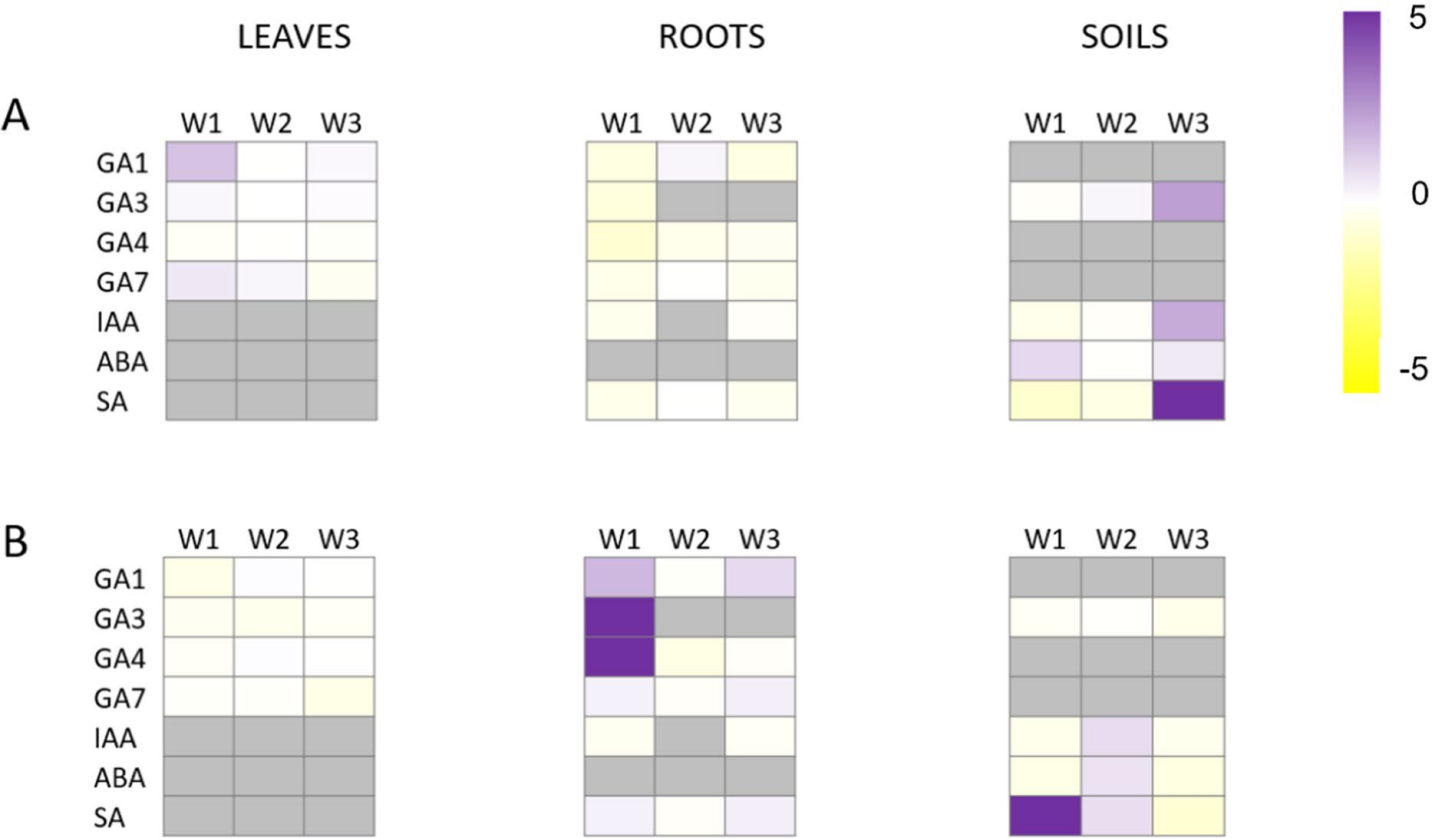
Phytohormonal changes after one week in plant tissues and soil samples of **A** plants irrigated with wastewater and spiked with 200 µg/L carbamazepine (CBZ) and climbazole (CLB) (Ls + PhACs), and **B** plants irrigated with wastewater and spiked with 200 µg/L CBZ and CLB and inoculated with T. harzianum (Ls + PhACs + Th). Fold-changes of indolacetic acid (IAA), gibberellins GA1, GA3, GA4 and GA7, abscisic acid (ABA) and salicylic acid (SA) in exposed plants (Ls + PhACs) are represented respect to control group in A and fold-changes in inoculated and exposed plants are represented respect to exposed plants (Ls + PhACs) in B, n = 3. These visualizations are descriptive; statistical significance of phytohormone profiles was assessed using multivariate analysis of variance (MANOVA) and is reported in the text

Gibberellins showed higher concentrations in root tissues, reaching up to 20 ng/g. Exposure to CBZ and CLB resulted in an increase in gibberellins GA1, GA3 and GA7 in leaves and a decrease of gibberellins GA1, GA3, GA4 and GA7 in roots (Fig. 4). In soils, only GA₃ was detected, and differences between control and exposed plants were observed only in the third week, showing an increase in exposed plants.

Gibberellins can be biosynthesized in meristematic root tissues, and may be released into the surrounding soil and lixiviated, becoming observable and quantifiable only after some time. In leaves, the increase in gibberellin concentrations appeared to be the highest during the first week, returning to control levels in the second and third weeks. A similar but inverse pattern was observed for gibberellin concentrations in roots, with a decrease in the first week followed by recovery in subsequent weeks.

Inoculated plants with *T. harzianum* showed similar concentrations than control plants (Fig. S2). In soils, only GA3 was detected. In control plants, GA3 concentration decreased from 0.89 to 0.39 ng/g. Exposure to PhACs had the opposite effect, increasing GA3 from 0.80 to 1.29 ng/g. Inoculation with T. harzianum was able to revert this trend and stabilize GA3 concentrations around 0.75 ng/g.

**Fig. 5:**
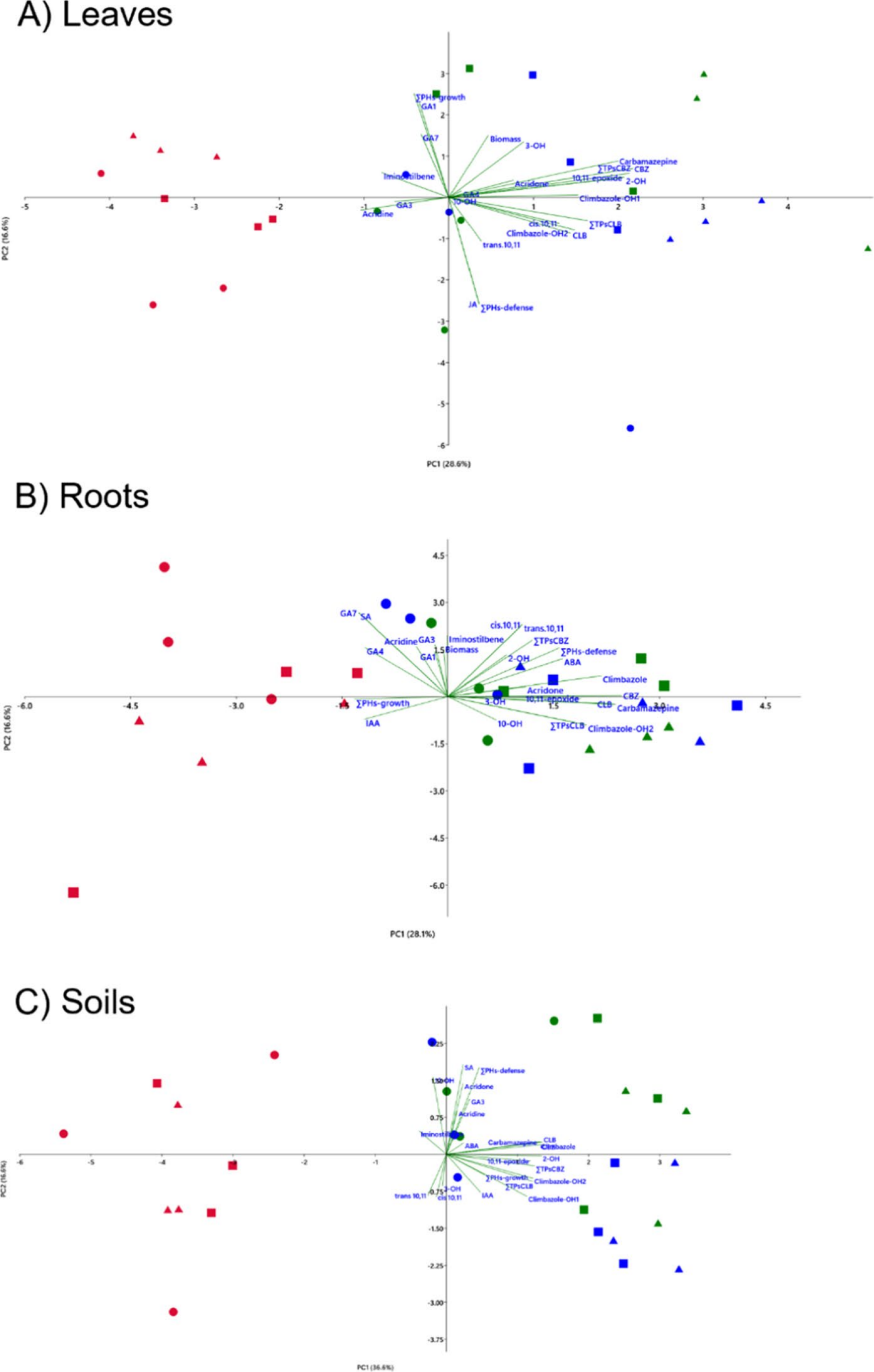
Scatterplot of leaf samples **A**, roots samples **B**, and soil samples **C** projected onto the first two principal components (PC1 and PC2). Each point represents an individual sample, positioned according to its multivariate profile, with distances between points reflecting differences in the underlying variables. Vectors represent the contribution of active variables. Colors denote treatment groups: red, plants irrigated with wastewater (Ls); green, plants irrigated with wastewater spiked with 200 µg/L CBZ and CLB (Ls + PhAC); and blue, plants inoculated with T. harzianum and irrigated with wastewater spiked with 200 µg/L CBZ and CLB (Ls + PhAC + Th). Symbols indicate sampling time: circles, week 1; squares, week 2; and triangles, week 3

IAA exhibited the highest concentrations among the PHs analyzed. It was prominently detected in both soil and root samples, with peak levels observed during the third week across all experimental groups (Fig. S2). IAA increased slowly towards the end of the experiment, with the highest values in control plants (97 ng/g) followed by exposed plants (85 ng/g) and by exposed and inoculated (67 ng/g). In roots, IAA concentrations were lower in exposed plants respect to controls (Fig. 5). The presence of *T. harzianum* did not change the distribution of IAA in roots. A distinct pattern emerged in soils, where IAA levels were lower in exposed plants during the first two weeks but increased in the third week (Fig. 4). However, in inoculated plants, a similar trend was observed. This suggests that *Trichoderma* inoculation did not significantly influence abiotic stress responses through IAA biosynthesis, which is typically associated with elongation and cell growth.

ABA was found only in soils with no big differences between groups and values ranging around 2 ng/g (Fig. 4). Like IAA, ABA did not appear to play a significant role in the *Trichoderma*-mediated response to xenobiotic exposure, as its levels showed no strong involvement in altering phytohormone-mediated stress responses (Fig. 4).

SA was detected in roots and soils. Whereas concentrations were decreasing in control soils, PhACs had the opposite effect, leading to an increase from the first week. Inoculation with *Trichoderma* reestablished the trend observed in control plants, with a decrease from the first to the last week. Like for gibberellins, the fungus was able to revert the original trend in SA concentrations found in control plants and disturbed by PhACs. SA is well known for its role in defense against both abiotic and biotic stresses, serving as an indicator of plant stress in response to external stressors. It has been demonstrated that other *Trichoderma* species, such as *T. longibrachiatum* TG1, cause an accumulation of SA, which raises antioxidant activity and the production of superoxide dismutase, peroxidase and catalase to combat the oxidative stress brought on by salinity (Boamah et al. 2022). Assuming that changes in phytohormonal levels obey plant defense responses against oxidative stress caused by exposure to organic pollutants, the application of the fungus appears to modulate this reaction by altering the pattern of PH biosynthesis and response. These changes are driven not only by defense PHs but also by PHs involved in growth and development, improving plant growth and biomass. Phytohormonal changes following fungal inoculation are accompanied by improved biomass production and a decrease in the uptake of PhACs and their TPs by plants. What remains unclear is whether the fungus directly modulated PH concentrations through molecular mechanisms (via fungal biosynthesis or induction of plant biosynthesis), or if this effect is instead a consequence of PhACs uptake and metabolism by the fungus in the soil environment. In the latter case, reduced availability of PhACs and their TPs for plant uptake would alleviate plant stress. Therefore, while the beneficial effects of *T. harzianum* application in agricultural soils for wastewater reuse have been demonstrated, further research is required to elucidate these mechanisms.

Overall, multivariate analysis revealed compartment-specific phytohormonal responses. Leaf phytohormone profiles remained globally stable across treatments and sampling weeks, indicating no coordinated hormonal shift induced by pharmaceutical exposure or *Trichoderma harzianum* inoculation (MANOVA, p > 0.05). In contrast, root phytohormone profiles were significantly influenced by sampling time (Pillai’s trace, p = 0.013), while treatment effects were not significant, suggesting that belowground hormonal dynamics were primarily driven by temporal rather than treatment-specific factors. Soil phytohormone profiles showed no significant multivariate effects of treatment or time (p > 0.05).

### Multivariate analysis (PCA) integrating biomass, contaminants, transformation products, and phytohormones

PCA analyses of leaves, roots, and soils captured 44–48% of the total variance along the first two axes (Fig. 5). In leaves (Fig. 5A), PC1 (28.6%) was primarily driven by CBZ, CLB, and their transformation products, separating control from pharmaceutical-treated samples, while PC2 (16.6%) reflected phytohormonal variation, highlighting treatment-dependent physiological responses, particularly in *Trichoderma*-inoculated plants. In roots (Fig. 5B), PC1 (28.1%) separated controls from pharmaceutical-treated plants and was influenced by both pharmaceuticals and defense-related phytohormones, whereas PC2 (15.2%) captured temporal hormonal dynamics. Soil PCA (Fig. 5C) showed PC1 (36.6%) separating control from pharmaceutical-amended soils, driven by pharmaceuticals and defense-associated phytohormones, while PC2 (16.6) reflected sampling time and hormonal variation, with *Trichoderma*-inoculated soils showing greater dispersion, suggesting modulation of soil biochemical dynamics rather than reduced pharmaceutical presence.

The MANOVAs confirmed these patterns, revealing significant effects of both sampling week and irrigation treatment in leaves (weeks: F_2,18_ = 3.6, *P*_perm_ < 0.01; treatments: F_2,18_ = 19.3, *P*_perm_ < 0.001), roots (weeks: F_2,18_ = 3.3, P_perm_ < 0.01; treatments: F_2,18_ = 8.2, *P*_perm_ < 0.001), and soils (weeks: F_2,18_ = 4.64, *P*_perm_ < 0.01; treatments: F_2,18_ = 17.84, *P*_perm_ < 0.001), with no significant week × treatment interactions.

In all samples, control leaves clustered on the negative side of PC1 and were associated with growth-related hormones, reflecting a non-stressed physiological status. PhAC-treated and PhACs + *Trichoderma* plants largely overlapped in the PCA space, indicating that pharmaceutical exposure was the main driver of variance. However, subtle shifts along PC2 suggest a modulation of specific physiological responses in the presence of *Trichoderma*.

### Methodological considerations and limitations

Several methodological aspects are relevant for the interpretation of the results obtained in this study. One such aspect concerns the use of autoclaved reclaimed wastewater. Autoclaving was applied to limit the contribution of wastewater-borne microorganisms and reduce uncontrolled microbial variability, particularly given the synthetic soil matrix. This approach may alter certain physicochemical properties of wastewater and potentially affect micropollutant stability. However, previous studies reported CBZ and CLB concentrations in Murviel WWTP effluents of 64 and 109 ng L⁻^1^, respectively (Manasfi et al., 2021), which are several orders of magnitude lower than the spiked concentrations used in this study (200 µg/L). It can therefore be reasonably assumed that potential thermal effects on background contaminants are unlikely to substantially influence the observed trends. While filter sterilization could better preserve chemical composition, its application at large volumes remains technically challenging and may introduce adsorption-related losses.

Beyond wastewater treatment, additional considerations related to fungal establishment should also be taken into account when interpreting the results. Although *T. harzianum* was applied as an inoculant, fungal colonization of the rhizosphere or plant tissues was not directly assessed in this study. Consequently, while the observed effects on plant biomass, pharmaceutical fate, and phytohormone profiles can be attributed to the presence of the fungus, it was not possible to establish quantitative relationships between these responses and fungal colonization level or biomass. The lack of direct colonization data limits mechanistic interpretation, particularly with respect to distinguishing rhizospheric versus endophytic contributions. Future studies combining chemical analyses with fungal quantification or localization approaches (e.g. plating, qPCR, or microscopy) would be valuable to better link fungal establishment with functional outcomes.

## Conclusions

This study explored the impact of pharmaceutical contamination on crops and the potential of *T. harzianum* as a remedial agent. The study found that while lettuce specimens were initially reduced in biomass production due to wastewater containing CBZ and CLB, *T. harzianum* had a positive impact on biomass accumulation during the nascent developmental phases. Trichoderma's ability to modulate the concentration gradients of these PhACs was observed in roots and leaves, with slightly elevated concentrations of CBZ within roots but diminished levels in leaves and soil substrates. This suggests that *T. harzianum* could influence the fate of PhACs within the rhizospheric milieu. The study also highlighted the complex interplay between pharmaceutical contamination and microbial interventions, with *T. harzianum* emerging as a promising mitigatory entity. By enhancing biomass production and shaping the spatial distribution of PhACs, *T. harzianum* represents a viable strategy for sustainable agriculture in wastewater-irrigated landscapes. These findings should be interpreted in light of methodological considerations related to wastewater treatment and fungal establishment. Further research integrating fungal establishment assessment and refined experimental approaches will be essential to elucidate the mechanistic basis and long-term robustness of Trichoderma-mediated pharmaceutical mitigation.

## Supplementary Information

Below is the link to the electronic supplementary material.

Supplementary file1 (DOCX 632 KB)

## Data Availability

No datasets were generated or analysed during the current study.
