# Supplementary material for: Trichoderma harzianum enhances lettuce biomass and modulates plant-soil emerging contaminant dynamics under reclaimed wastewater irrigation
Source: Biodegradation. 2026 Apr 15;37(3):70. doi: 10.1007/s10532-026-10291-0 (PMC13083496; doi:10.1007/s10532-026-10291-0)
Supplement: Supplementary file 1 — Supplementary file1 (DOCX 632 KB) [file 10532_2026_10291_MOESM1_ESM.docx]

***Trichoderma harzianum* enhances lettuce biomass and modulates plant-soil emerging contaminant dynamics under reclaimed wastewater irrigation**

Monica Brienza^1*^ , Juan Manuel Peña-Herrera^2,3^, Vincenzo Trotta^4^, Serge Chiron^2^, Andrés Sauvêtre^5*^

^1^ Dipartimento di Scienze di Base ed Applicate, Università degli Studi della Basilicata,Via dell’Ateno 10, 85100 Potenza.

^2^ HydroSciences Montpellier, University of Montpellier, IMT Mines Alès, IRD, CNRS, 34 Ave Charles Flahault 34093 Montpellier cedex 5, France.

^3^Laboratorio Bioanalitics, Vicerrectoría de investigaciones, Universidad del Valle, Cali-Colombia.

^4^Dipartimento di Scienze Agrarie, Forestali, Alimentari e Ambientali, Università degli Studi della Basilicata, Via dell’Ateno 10, 85100 Potenza.

^5^HydroSciences Montpellier, University of Montpellier, IMT Mines Alès, IRD, CNRS, Alès, France

*Corresponding author: monica.brienza@unibas.it, +39 3469474178;

` [andre.sauvetre@mines-ales.fr](mailto:andre.sauvetre@mines-ales.fr), +33 466782765

Table 1 : Daily irrigation volumes (Vol.) applied to the soil, the accumulated volume of wastewater (WW) irrigated over time, and the corresponding cumulative mass of pharmaceutical active compounds (PhAC) delivered. The PhAC concentration in the spiked wastewater was 200 ng/mL. The harvest was performed on Days 8 (1 week), 15 (2 weeks) and 22 (3 weeks).

| Day | Volume (mL) | Wastewater cumulative volume (mL) | PhAC cumulative mass (ng) |
| --- | --- | --- | --- |
| 1 | 90 | 90 | 18000 |
| 5 | 90 | 180 | 36000 |
| 8 | 100 | 280 | 56000 |
| 11 | 90 | 370 | 74000 |
| 15 | 90 | 460 | 92000 |
| 18 | 100 | 560 | 112000 |
| 21 | 100 | 660 | 132000 |
| 22 |  |  |  |


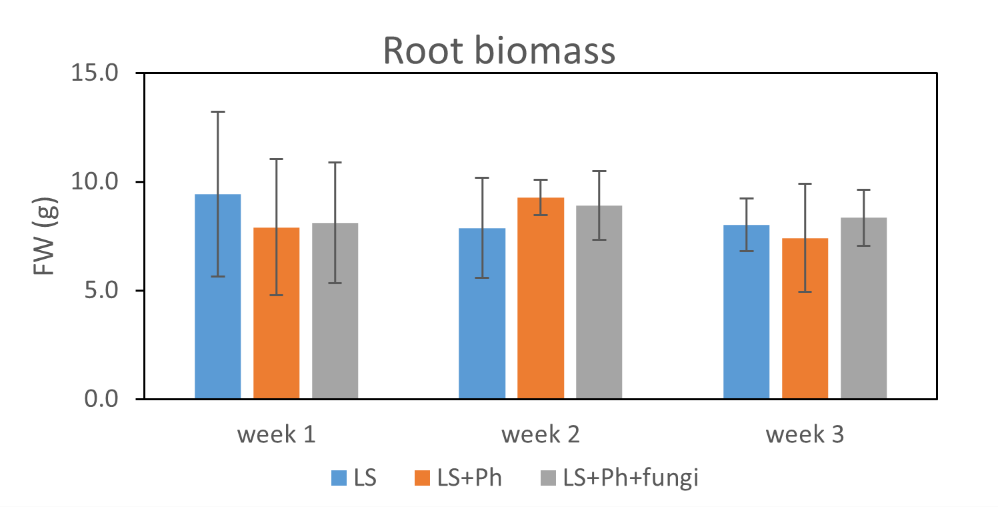


Figure S1 : Root biomass expressed as fresh weight (FW, g) of lettuce control plants (Ls), lettuce plants irrigated with wastewater spiked with 200 µg/L CLB and CBZ (Ls + PhAC) and lettuce plants inoculated with T. harzianum and irrigated with wastewater spiked with 200 µg/L CLB and CBZ (Ls + PhAC + Th) over three weeks. Data are presented as mean ± standard deviation.


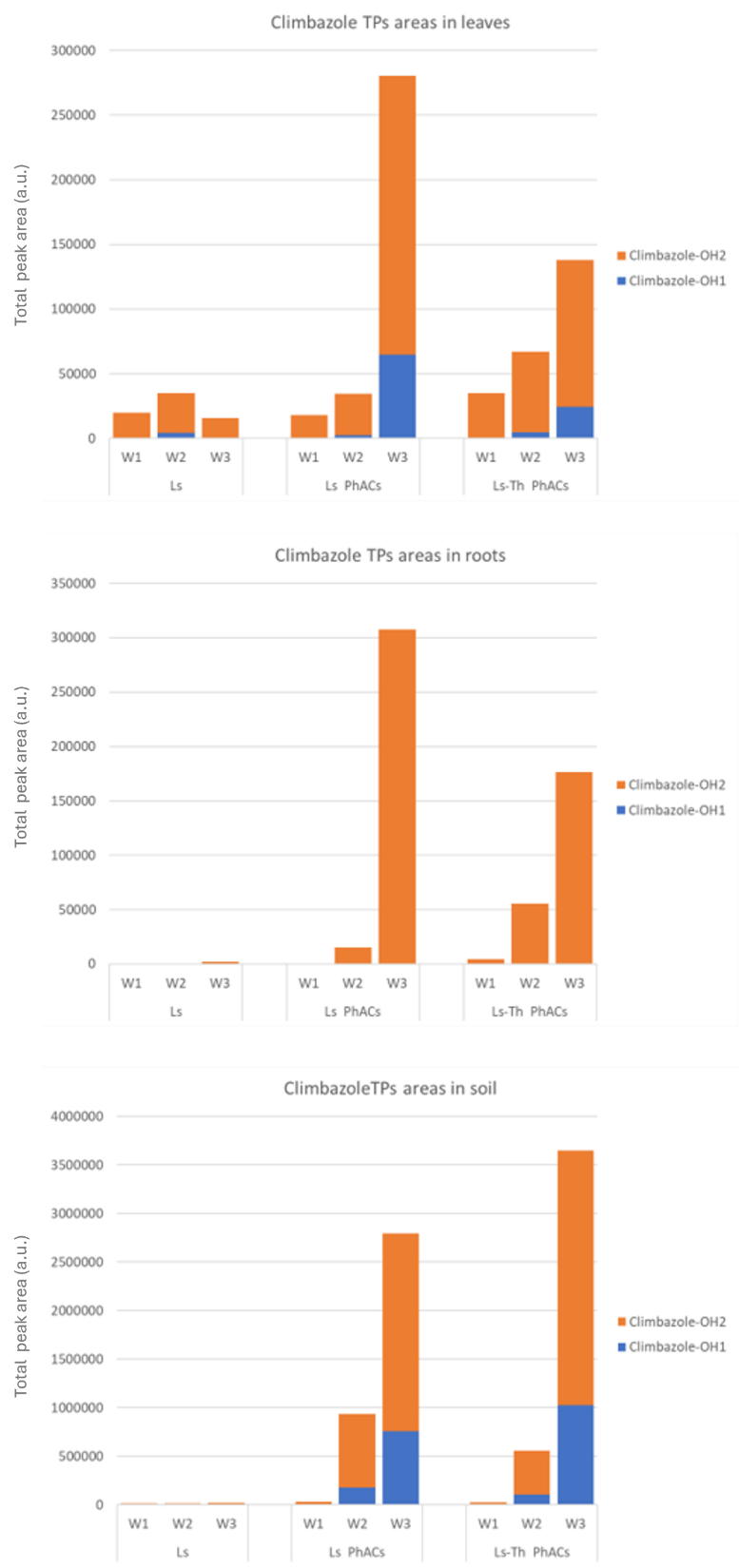


Figure S2: Distribution of CLB transformation products after three weeks of irrigation in plant tissues and soils of control plants (Ls), plants irrigated with wastewater spiked with 200 µg/L CLB and CBZ (Ls + PhAC) and plants inoculated with T. harzianum and irrigated with wastewater spiked with 200 µg/L CLB and CBZ (Ls + PhAC + Th). Results are the average of three biological replicates and are expressed as LC-MS/MS peak areas.


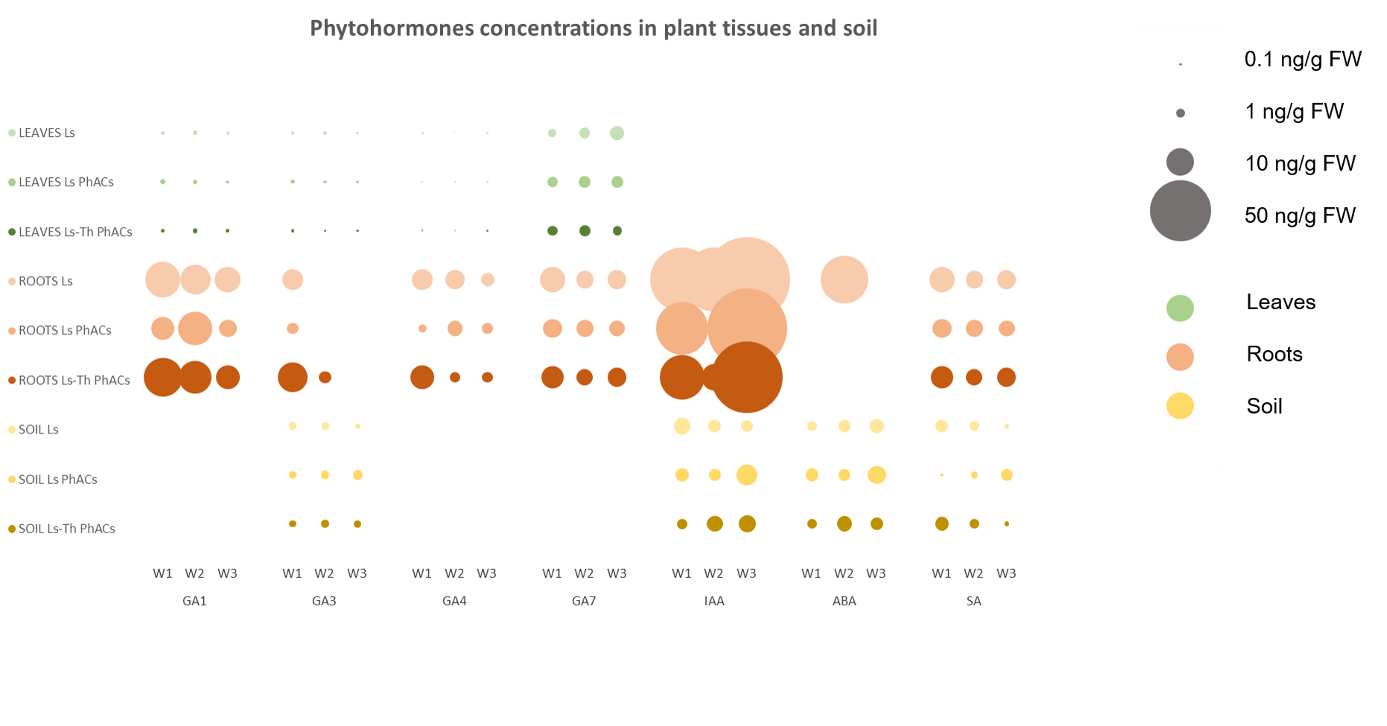


Figure S3 : Concentrations of the PHs gibberellins GA1, GA3, GA4, and GA7, indolacetic acid (IAA), abscisic acid (ABA), and salicylic acid (SA) in soils and plant tissues (roots and leaves) in plants irrigated with wastewater (Ls), plants irrigated with wastewater (Ls), plants irrigated with wastewater spiked with 200 µg/L CLB and CBZ (Ls + PhACs), and plants irrigated with wastewater spiked with 200 µg/L CLB and CBZ and inoculated with T. harzianum (Ls + PhACs + Th), n = 3. These visualizations are descriptive; statistical significance of phytohormone profiles was assessed using multivariate analysis of variance (MANOVA) and is reported in the text.

Table 2: Average peak areas of CBZ transformation products (∑TPsCBZ) and total CBZ-related compounds (CBZ + TPs) in leaves, roots, and soil under different treatments across three weeks (Ls : control plants (Ls) ; Ls+PhACs : plants irrigated with wastewater spiked with 200 µg/L CLB and CBZ ; and Ls+PhACs+Th : plants inoculated with T. harzianum and irrigated with wastewater spiked with 200 µg/L CLB and CBZ). The percentage of ∑TPsCBZ represents the relative contribution of transformation products to the total signal. STD indicates standard deviation (n=3).

| **Compartment** | **Treatment** | **Week** | **Average Peak Area** | | **∑TPsCBZ (in %)** | **STD** |
| --- | --- | --- | --- | --- | --- | --- |
|  |  |  | **∑TPsCBZ** | **TOTAL (CBZ + TPs)** |  |  |
| Leaves | Ls | 1 | 91911 | 140321 | 72.18 | 24.87 |
|  |  | 2 | 59866 | 320657 | 18.55 | 1.51 |
|  |  | 3 | 43952 | 544637 | 8.47 | 2.85 |
|  | Ls+PhACs | 1 | 344590 | 23315139 | 1.42 | 0.72 |
|  |  | 2 | 1588143 | 77816564 | 2.08 | 0.41 |
|  |  | 3 | 7498861 | 169705956 | 4.42 | 1.31 |
|  | Ls+PhACs+Th | 1 | 383818 | 23466258 | 1.68 | 0.37 |
|  |  | 2 | 1424136 | 75449310 | 1.84 | 0.35 |
|  |  | 3 | 5067786 | 129117288 | 3.92 | 0.31 |
| Roots | Ls | 1 | 33119 | 54388 | 63.57 | 27.99 |
|  |  | 2 | 35511 | 444112 | 6.95 | 3.08 |
|  |  | 3 | 40903 | 112780 | 39.58 | 27.78 |
|  | Ls+PhACs | 1 | 36487 | 13905571 | 0.26 | 0.11 |
|  |  | 2 | 50431 | 31770965 | 0.14 | 0.09 |
|  |  | 3 | 142612 | 32171165 | 0.46 | 0.61 |
|  | Ls+PhACs+Th | 1 | 34785 | 14670586 | 0.24 | 0.08 |
|  |  | 2 | 50244 | 33120956 | 0.15 | 0.06 |
|  |  | 3 | 42296 | 35678121 | 0.12 | 0.01 |
| Soil | Ls | 1 | 112188 | 365566 | 30.67 | 3.71 |
|  |  | 2 | 114292 | 1843593 | 7.06 | 2.62 |
|  |  | 3 | 128474 | 884866 | 14.26 | 4.37 |
|  | Ls+PhACs | 1 | 248154 | 89783488 | 0.28 | 0.12 |
|  |  | 2 | 902026 | 140023552 | 0.64 | 0.02 |
|  |  | 3 | 1583774 | 181754054 | 0.89 | 0.38 |
|  | Ls+PhACs+Th | 1 | 228184 | 84975070 | 0.27 | 0.06 |
|  |  | 2 | 780910 | 133400453 | 0.59 | 0.09 |
|  |  | 3 | 1749323 | 165711494 | 1.05 | 0.07 |
